# *Gynoxys hallii* Hieron., *Gynoxys calyculisolvens* Hieron., and *Gynoxys azuayensis* Cuatrec. Essential Oils—Chemical and Enantioselective Analyses of Three Unprecedented Volatile Fractions from the Ecuadorian Biodiversity

**DOI:** 10.3390/plants14050659

**Published:** 2025-02-21

**Authors:** Yessenia E. Maldonado, María del Carmen Rodríguez, María Emilia Bustamante, Stefanny Cuenca, Omar Malagón, Nixon Cumbicus, Gianluca Gilardoni

**Affiliations:** 1 Programa de Doctorado en Química, Universidad Técnica Particular de Loja (UTPL), Calle Paris s/n y Praga, Loja 110107, Ecuador; yemaldonado2@utpl.edu.ec; 2 Carrera de Bioquímica y Farmacia, Universidad Técnica Particular de Loja (UTPL), Calle Paris s/n y Praga, Loja 110107, Ecuador; mdrodriguez15@utpl.edu.ec (M.d.C.R.); mebustamante8@utpl.edu.ec (M.E.B.); smcuenca9@utpl.edu.ec (S.C.); 3 Departamento de Química, Universidad Técnica Particular de Loja (UTPL), Calle Paris s/n y Praga, Loja 110107, Ecuador; omalagon@utpl.edu.ec; 4 Departamento de Ciencias Biológicas y Agropecuarias, Universidad Técnica Particular de Loja (UTPL), Calle Paris s/n y Praga, Loja 110107, Ecuador; nlcumbicus@utpl.edu.ec

**Keywords:** Asteraceae, enantiomers, chiral separation, gas chromatography, mass spectrometry

## Abstract

The present study is the first report on the chemical and enantiomeric compositions of essential oils from the Ecuadorian species *Gynoxys hallii* Hieron., *Gynoxys calyculisolvens* Hieron., and *Gynoxys azuayensis* Cuatrec. All the volatile fractions presented a sesquiterpene-based chemical profile, typical of other volatile fractions from this genus. Both qualitative (GC-MS) and quantitative (GC-FID) chemical analyses were carried out on two stationary phases of different polarity (non-polar and polar). The main constituents of *G. hallii* essential oil on the two columns, respectively, were α-pinene (33.6–31.5%), (*E*)-β-caryophyllene (6.2–6.4%), germacrene D (35.7–38.3%), and bicyclogermacrene (3.8–4.0%). In *G. calyculisolvens*, the major compounds were α-pinene (11.2–11.0%), *p*-cymene (4.0–3.7%), α-copaene (3.6–3.7%), (*E*)-β-caryophyllene (8.1–8.3%), germacrene D (20.8–22.0%), and germacrene D-4-ol (8.4–8.6%). Finally, the main components of *G. azuayensis* were α-pinene (4.5–4.1%), germacrene D (14.1–12.4%), bicyclogermacrene (2.6–3.0%), tridecanal (6.4–6.2%), and spathulenol (7.8–7.1%). Furthermore, enantioselective analyses were conducted on the three volatile fractions, using two stationary phases based on β-cyclodextrins. As a result, twelve chiral components were investigated, detecting both enantiomerically pure compounds and scalemic mixtures with various enantiomeric excess.

## 1. Introduction

According to the United Nations, Ecuador has been defined as a megadiverse country, whose outstanding biodiversity includes thousands of phytochemically unstudied botanical species [1,2,3]. For this reason, our group has been studying the secondary metabolites of native and endemic Ecuadorian plants for more than twenty years, with the aim of discovering new natural products of pharmaceutical and biochemical interest [4]. In recent years, we have especially focused on the description of unprecedented essential oils (EOs), describing their chemical and enantiomeric compositions, olfactometric profiles, and biological activities [5]. With these premises, an unfunded project about the volatile fractions of genus *Gynoxys* in southern Ecuador is currently in progress, based on the chemical compositions and enantiomeric profiles of the EOs from this taxon. The focus of this project is strictly academic and chemotaxonomic due to the low distillation yields that usually characterise these EOs. A total of twelve unprecedented species were selected in the Province of Loja, Ecuador: *Gynoxys miniphylla* Cuatrec., *Gynoxys laurifolia* (Kunth) Cass., *Gynoxys rugulosa* Muschl., *Gynoxys buxifolia* (Kunth) Cass., *Gynoxys cuicochensis* Cuatrec., *Gynoxys sancti-antonii* Cuatrec., *Gynoxys szyszylowiczii* Hieron., *Gynoxys reinaldii* Cuatrec., *Gynoxys pulchella* (Kunth) Cass., *Gynoxys hallii* Hieron., *Gynoxys calyculisolvens* Hieron., and *Gynoxys azuayensis* Cuatrec. The first nine species have already been studied and their EOs published, whereas *G. hallii*, *G. calyculisolvens*, and *G. azuayensis* are the object of the present research [6,7,8,9,10,11,12].

Botanically, the genus *Gynoxys* Cass. (Asteraceae) is an endemic taxon of the Andean region, diffused from Venezuela to Bolivia, with Ecuador being the country with the highest number of collected specimens [13]. Among the Ecuadorian species, *G. hallii*, *G. calyculisolvens*, and *G. azuayensis* have been reported in the Province of Loja. According to the Catalogue of the Vascular Plants of Ecuador, *G. hallii* is an endemic treelet growing between 2500–3500 m above sea level and described in the provinces of Azuay, Cañar, Carchi, Chimborazo, Cotopaxi, Imbabura, Pichincha, and Tungurahua. In the same source, *G. calyculisolvens* is a native shrub growing between 2000–3500 m above sea level (m a.s.l.) and occurring in the provinces of Loja and Morona-Santiago. Finally, *G. azuayensis* is an endemic tree growing in the range 2500–3500 m a.s.l. that has been observed in the provinces of Azuay and Loja [14]. None of these three species are reported with botanical synonyms or relevant traditional use. To the best of the authors’ knowledge, the present study is the first description of EOs obtained from *G. hallii*, *G. calyculisolvens*, and *G. azuayensis*.

## 2. Results

### 2.1. Chemical Composition of the EOs

**Figure 1:**
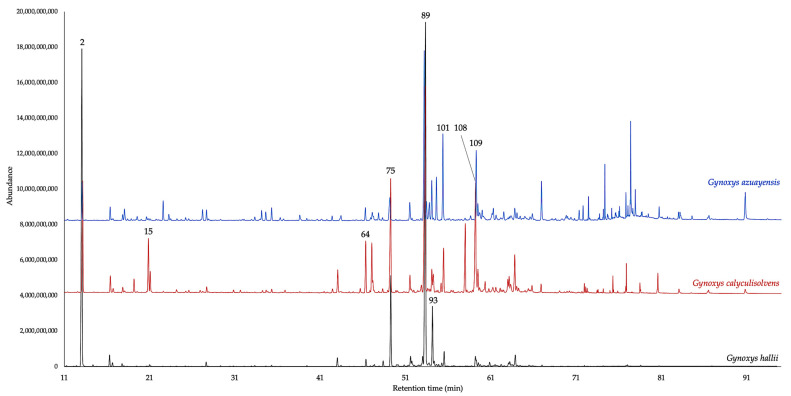
Compared GC–MS profiles of *G. hallii* (black), *G. calyculisolvens* (red), and *G. azuayensis* (blue) EOs on a 5% phenyl methylpolysiloxane stationary phase. The numbers refer to column N in Table 1.

**Figure 2:**
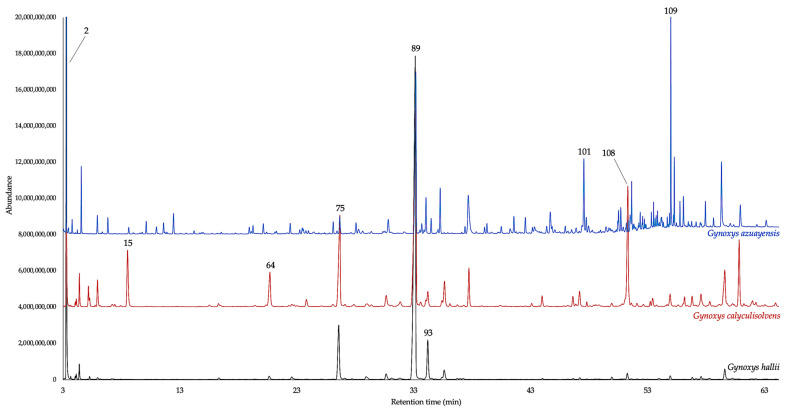
Compared GC–MS profiles of *G. hallii* (black), *G. calyculisolvens* (red), and *G. azuayensis* (blue) EOs on a polyethylene glycol stationary phase. The numbers refer to column N in Table 1.

**Figure 3:**
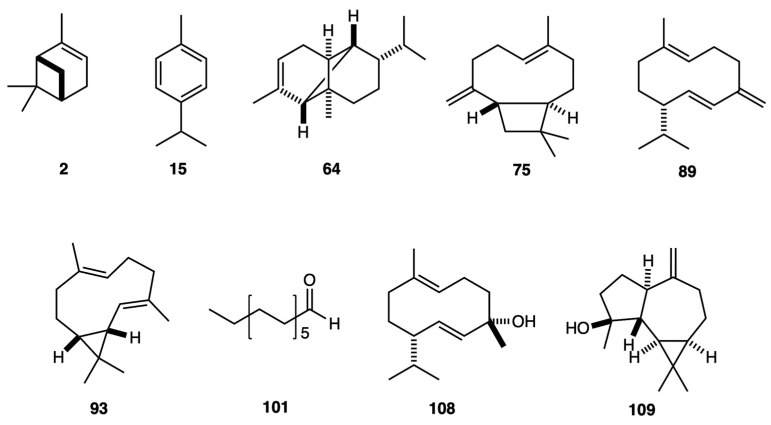
Molecular structures of the major compounds whose abundance is ≥3.0% on at least one column in at least one EO. According to Table 1, these molecules are α-pinene (**2**), *p*-cymene (**15**), α-copaene (**64**), (*E*)-β-caryophyllene (**75**), germacrene D (**89**), bicyclogermacrene (**93**), tridecanal (**101**), germacrene D-4-ol (**108**), and spathulenol (**109**).

The leaves of *G. hallii*, *G. calyculisolvens*, and *G. azuayensis* afforded, by analytical distillation, three EOs that, respectively, yielded 0.69 ± 0.105%, 0.08 ± 0.008%, and 0.02 ± 0.002% by weight of dry plant material. A total of 169 compounds were identified and quantified on at least one of the two employed columns. The quantified components, separately considered on the non-polar and polar stationary phase, respectively, and expressed as oil mass percent, corresponded to 93.2–92.7% for *G. hallii*, 94.9–93.8% for *G. calyculisolvens*, and 94.2–89.3% for *G. azuayensis*. All the volatile fractions were dominated by sesquiterpenes and sesquiterpenoids that, considered as a whole, corresponded to 56.8–58.5% for *G. hallii*, 60.7–59.8% for *G. calyculisolvens*, and 45.9–39.7% for *G. azuayensis*. In the EO of *G. azuayensis*, an important heavy aliphatic fraction is also present (about 30%), as typical of other species from this genus. On the other hand, in *G. hallii* and *G. calyculisolvens*, this fraction is absent or represented in a very low amount. Finally, a relevant monoterpene fraction, dominated by α-pinene, can also be observed in the three oils, reaching the maximum amount in *G. hallii* and the minimum value in *G. azuayensis*. Main constituents of *G. hallii* EO (≥3.0% on at least one column) were α-pinene (peak 2), (*E*)-β-caryophyllene (peak 75), germacrene D (peak 89), and bicyclogermacrene (peak 93). In *G. calyculisolvens* EO, the major compounds were α-pinene (peak 2), *p*-cymene (peak 15), α-copaene (peak 64), (*E*)-β-caryophyllene (peak 75), germacrene D (peak 89), and germacrene D-4-ol (peak 108). Finally, the main components of *G. azuayensis* EO were α-pinene (peak 2), germacrene D (peak 89), bicyclogermacrene (peak 93), tridecanal (peak 101), and spathulenol (peak 109). The detailed qualitative and quantitative compositions of these EOs are reported in Table 1, whereas the gas chromatographic profiles are represented in Figure 1 and Figure 2. The molecular structures of the nine major compounds are represented in Figure 3.

### 2.2. Enantioselective Analyses of the EOs

The enantioselective analyses of the three EOs permitted to evaluate the enantiomeric excess of a total of 12 enantiomeric pairs, using one of two available chiral selectors, depending on the chromatographic resolution. In this way, (1*S*,5*S*)-(−)-α-pinene, (*R*)-(−)-α-phellandrene, (*R*)-(−)-β-phellandrene, and (1*S*,2*R*,6*R*,7*R*,8*R*)-(+)-α-copaene resulted as enantiomerically pure in all the EOs where they were present. On the other hand, (*R*)-(−)-terpinen-4-ol and (*S*)-(−)-germacrene D were enantiomerically pure in *G. azuayensis* and *G. hallii*, respectively, but they were part of scalemic mixtures in the other species. Finally, all the others analysed chiral metabolites produced scalemic mixtures in all the volatile fractions where they were detected. The detailed results of the enantioselective analyses are shown in Table 2.

## 3. Discussion

**Figure 4:**
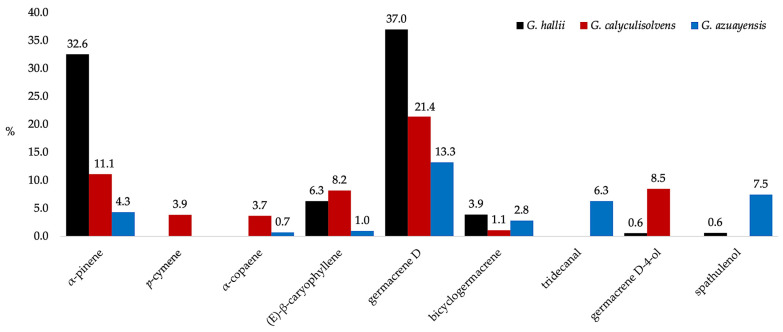
Compared abundance of major compounds (≥3.0% in at least one oil) in the EOs of *G. hallii* (black), *G. calyculisolvens* (red), and *G. azuayensis* (blue). Abundances correspond to the mean values of the quantitative results on both columns.

The chemical composition of these three EOs presented the typical profile of most *Gynoxys* volatile fractions. Especially in *G. azuayensis* and, to a lesser extent in *G. calyculisolvens*, three characteristic groups of metabolites can be observed: a monoterpene fraction dominated by α-pinene (**2**); a sesquiterpene fraction with germacrene D (**89**) as the main component; and a heavy aliphatic fraction composed of alkanes and alkenes. Such a profile is very evident in *G. rugulosa*, *G. szyszylowiczii*, and *G. pulchella* but less important in *G. laurifolia*, *G. sancti-antonii*, and *G. cuicochensis* [7,8,10,11]. In the case of *G. hallii*, the heavy aliphatic fraction is practically absent, which was previously observed in *G. miniphylla* and *G. buxifolia* [6,9]. A comparison among the major constituents (≥3.0% in at least one oil) of *G. hallii*, *G. calyculisolvens*, and *G. azuayensis* volatile fractions is represented in Figure 4. It can be observed that α-pinene (**2**), (*E*)-β-caryophyllene (**75**), germacrene D (**89**), and bicyclogermacrene (**93**) are common to the three species, this pattern being characteristic of *Gynoxys spp.* Other compounds are abundant constituents of only one species, such as *p*-cymene (**15**) in *G. calyculisolvens* and tridecanal (**101**) and spathulenol (**109**) in *G. azuayensis*.

**Figure 5:**
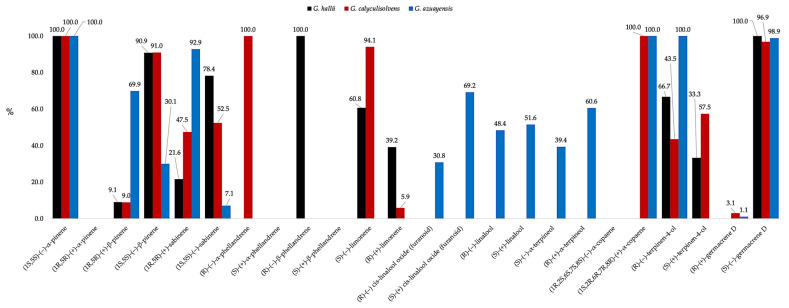
Compared enantiomeric composition of some chiral compounds in the EOs of *G. hallii* (black), *G. calyculisolvens* (red), and *G. azuayensis* (blue).

For what concerns the enantiomeric compositions of *G. hallii*, *G. calyculisolvens*, and *G. azuayensis* EOs (see Figure 5), three characteristics can be observed that are quite common to other species of this genus. First of all, a similar pattern can be observed for (1*S*,5*S*)-(–)-α-pinene and (S)-(–)-germacrene D. These compounds are in fact enantiomerically pure or close to optical purity in the three EOs, confirming a situation already seen before. Secondly, the oxygenated monoterpenoids are present as scalemic mixtures, with a quite low enantiomeric excess that makes their composition relatively close to a racemate. Finally, it can be observed that α-pinene and β-pinene, despite deriving from the common pinyl cation, exhibited a different enantiomeric excess. As a consequence, since the same absolute configuration should be expected for these two monoterpenes, the existence of some enantiospecific reaction could explain the loss of (1*R*,5*R*)-(+)-α-pinene [113].

Among the main components of *G. hallii*, *G. calyculisolvens*, and *G. azuayensis* EOs, five compounds were the most representative constituents. In fact, each one of the following metabolites, α-pinene (**2**), (*E*)-β-caryophyllene (**75**), germacrene D (**89**), germacrene D-4-ol (**108**), and spathulenol (**109**), reached at least 7% of the oil mass in at least one species, suggesting a possible relevant contribution to the properties of the corresponding volatile fractions in terms of biological activities.

According to literature, α-pinene (**2**), one of the most common monoterpenes, exhibited a wide range of biological capacities. For instance, it is known for its antibacterial potential, particularly against methicillin-resistant *Staphylococcus aureus* (MRSA). It also exhibited antifungal activity and has been found to be more effective than clotrimazole against *Candida* spp. This monoterpene also displayed anti-inflammatory properties, reducing the expression of inflammatory mediators like TNF-α and IL-1β. Additionally, it has demonstrated neuroprotective effects, enhancing learning and memory function in cases of scopolamine-induced memory deficit. Further studies have revealed its potential as an anticonvulsant and anti-leishmania agent. In addition, there is evidence of different biological activities exhibited by the enantiomers of α-pinene, highlighting the importance of monitoring enantiomeric distributions [114].

(*E*)-β-Caryophyllene (BCP, **75**) is a natural bicyclic sesquiterpene, perhaps the most common sesquiterpene in plants, and has demonstrated a wide range of biological activities. In fact, BCP binds to CB2 cannabinoid receptors and interacts with peroxisome-proliferator-activated receptors (PPARs). Because of these interactions, BCP exhibited anti-inflammatory effects by reducing the production of pro-inflammatory mediators, including TNF-α, IL-1β, IL-6, and NF-κB. Additionally, BCP showed neuroprotective activity in models of Alzheimer’s disease, where it decreased NO synthesis and reduced the activation of astrocytes and microglia [115,116].

Germacrene D (**89**) is another natural sesquiterpene that acts as a key intermediate in the biosynthesis of many other metabolites from the same family. Furthermore, it has been shown to influence the behaviour of moths, for example, increasing attraction and oviposition on mated females of *Heliotis virescens*. In fact, germacrene D receptor neurons have been identified in the moths *Helicoverpa armigera*, *H. assulta*, and *H. virescens*, where an enantiospecific response for the laevorotatory enantiomer of germacrene D has been demonstrated [117,118].

Germacrene D-4-ol (**108**) is an oxygenated derivative of germacrene D, and few studies have been reported so far about its biological activities. This sesquiterpenoid is abundant in the leaves of *Piper corcovadensis*, from which it can be obtained by steam distillation or solvent extraction. Both the isolated compound and the oil exhibited significant larvicidal activity against the mosquito *Aedes aegypti*, with LC_50_ values of 18.23 ± 1.19 ppm and 6.71 ± 0.16 ppm, respectively. Additionally, both demonstrated oviposition deterrent activity at various concentrations. Molecular docking analysis suggested that germacrene-D-4-ol exerts its oviposition deterrent effect by interacting with the odorant-binding protein 1 (OBP1) of *A. aegypti* [119].

Finally, spathulenol (**109**), a major component of G. *hallii* EO, must be mentioned. It is a sesquiterpene alcohol quite common in many volatile fractions, but little investigated from the pharmacological point of view. One of the few studies about it dealt on the EO of *Psidium guineense* (EOPG). A total of 38 compounds were identified, with spathulenol (PG-1) being the most abundant (80.7%). Both EOPG and PG-1 demonstrated antioxidant activity in DPPH, ABTS, and MDA assays. They also exhibited anti-inflammatory effects in mice by significantly inhibiting carrageenan-induced paw oedema, pleural cell migration, and protein exudation. Furthermore, EOPG and PG-1 were evaluated for antiproliferative activity against various cancer cell lines, particularly against OVCAR-3 (ovarian cancer). Furthermore, both EOPG and PG-1 displayed moderate antimycobacterial activity against *Mycobacterium tuberculosis* [120].

Concerning some possible practical applications and based on this bibliographic information, the three EOs from *G. hallii*, *G. calyculisolvens*, and *G. azuayensis* would be expected to be anti-inflammatory products if their relatively low distillation yields did not prevent a sustainable exploitation of the wild species. Similarly, the high content of the laevorotatory germacrene D would suggest a possible use as insect attractants. On the other hand, in the only case of *G. calyculisolvens*, the latter property could be associated to a larvicidal capacity due to the presence of germacrene D-4-ol in a relatively high amount. Naturally, the same applications could be suggested for each major compound in its pure form.

## 4. Materials and Methods

### 4.1. Plant Materials

The leaves of the three species were gathered in the Province of Loja, Ecuador, within approximately 200 m of the following coordinates: 03°31′22″ S, 79°23′59″ W for *G. hallii* (elevation: 2810 m), 03°43′11″ S, 79°20′04″ W for *G. calyculisolvens* (elevation: 3490 m), and 03°59′26″ S, 79°09′39″ W for *G. azuayensis* (elevation: 2590 m). The botanical identification of the specimens was carried out by one of the authors (N.C.) based on reference samples with barcodes 01826294 (*G. hallii*), 01826134 (*G. calyculisolvens*), and 01826026 (*G. azuayensis*) and preserved at the National Museum of Natural History, Smithsonian Institution, Washington, DC, USA.

Following collection, a voucher for each species was also deposited at the herbarium of Universidad Técnica Particular de Loja, assigned the codes 14678 (*G. hallii*), 14673 (*G. calyculisolvens*), and 14816 (*G. azuayensis*). These collections were conducted in accordance with Ecuadorian law and authorised by the Ministry of Environment, Water, and Ecological Transition of Ecuador (MAATE) under permit code MAATE-DBI-CM-2022-0248.

On the same day they were collected, the plant materials were dried at 35 °C for 48 h and then stored in a cool, dark place until the distillation process.

### 4.2. Distillation of the EOs

The dry leaves were steam distilled using an analytical method, as described in the literature, with a modified Dean–Stark apparatus [8]. In this procedure, the plant material was distilled for four hours over 2 mL of cyclohexane, containing 1.4 mg of n-nonane as an internal standard. This process was carried out four times for each species, yielding samples in cyclohexane solutions that could be directly injected into the GC. Both n-nonane and cyclohexane were obtained from Merck (Sigma–Aldrich, St. Louis, MO, USA). The weights of dry leaves distilled in each repetition were as follows: 81.5 g, 84.5 g, 84.6 g, and 84.6 g for *G. hallii*; 100.8 g, 100.7 g, 101.1 g, and 109.7 g for *G. calyculisolvens*; and 57.8 g, 57.7 g, 46.6 g, and 50.0 g for *G. azuayensis*. Following distillation, all cyclohexane solutions were permanently stored at −14 °C.

### 4.3. Qualitative Analyses of the EOs (GC-MS)

The qualitative analyses were carried out using a gas chromatograph (GC) model Trace 1310, supplied by Thermo Fisher Scientific (Waltham, MA, USA). The GC was coupled with a single quadrupole mass spectrometer (MS) model ISQ 7000, also obtained from the same provider. The electron impact ion source was set at 70 eV, with the mass analyser operating in SCAN mode over a mass range of 40–400 *m*/*z*. The ion source and quadrupole were heated to 250 °C, while the transfer line and injector were set to 230 °C. The samples were injected in split mode (40:1), introducing 1 μL of cyclohexane solution. Elution was performed according to the following thermal programme: 50 °C for 10 min, followed by an initial temperature gradient of 2 °C/min up to 170 °C and a second gradient of 10 °C/min up to 230 °C, which was maintained for 20 min. The carrier gas used was helium, maintained at a constant flow rate of 1 mL/min (Indura, Guayaquil, Ecuador). All essential oils (EOs) were analysed using two stationary phases with different polarities: one based on 5% phenyl-methylpolysiloxane (TR-5, non-polar) and the other one on polyethylene glycol (TR-WAX, polar). Both columns measured 30 m in length, with an internal diameter of 0.25 mm and a film thickness of 0.25 μm, and were purchased from Thermo Fisher Scientific (Waltham, MA, USA). The EO constituents were identified by comparing their linear retention indices (LRIs) and mass spectra with data available in the literature (see Table 1). The LRIs were calculated according to Van den Dool and Kratz, using a series of *n*-alkanes ranging from C_9_ to C_26_ (Sigma–Aldrich, St. Louis, MO, USA) [121].

### 4.4. Quantitative Analyses of the EOs (GC-FID)

The quantitative analyses were conducted using the same instrument, columns, thermal programme, carrier gas flow, injector temperature, and injection volumes as those used for the qualitative analyses. However, a flame ionisation detector (FID) was used instead of an MS, and the split ratio was set to 10:1. The components of the essential oils (EOs) were quantified by multiplying each peak area by a relative response factor (RRF), calculated based on combustion enthalpy, as described by Alain Chaintreau [122,123]. The corrected peak areas were then applied, for each column, to a six-point calibration curve, using isopropyl caproate as the calibration standard and *n*-nonane as the internal standard. The standard dilutions were prepared following the previously described methods in the literature, consistently achieving correlation coefficients greater than 0.998 [124]. The calibration standard was synthesised in the authors’ laboratory and purified to 98.8% (GC-FID).

### 4.5. Enantioselective Analyses of the EOs

The relative abundance of 12 enantiomeric pairs was determined using GC-MS, employing the same instrument, settings, and injection parameters as in the qualitative analyses, except for the carrier gas flow, which was maintained at a constant pressure of 70 kPa. The separations were performed using two enantioselective capillary columns, incorporating 2,3-diacetyl-6-*tert*-butyldimethylsilyl-β-cyclodextrin (DAC) and 2,3-diethyl-6-*tert*-butyldimethylsilyl-β-cyclodextrin (DET) as chiral selectors, both obtained from Mega (Milan, Italy). Elution was carried out following this thermal gradient: 50 °C for 1 min, followed by a temperature gradient of 2 °C/min up to 220 °C, which was then held for 10 min. The enantiomers were identified based on their mass spectra and by injecting enantiomerically pure standards under the same conditions. The LRIs of the enantiomers were also calculated for both columns according to Van den Dool and Kratz, using the same *n*-alkane mixture mentioned in Section 4.3. The choice of chiral selectors was based on the quality of separation achieved for each enantiomeric pair.

## 5. Conclusions

The leaves of *Gynoxys hallii* Hieron., *Gynoxys calyculisolvens* Hieron., and *Gynoxys azuayensis* Cuatrec. produced an EO with a yield by weight of 0.69%, 0.08%, and 0.02%, respectively. On the one hand, the yield of *G. hallii* was sufficiently high to be consistent with a possible practical application; on the other hand, the three EOs were characterised by the typical chemical profile of many other volatile fractions from this genus. Based on literature, these chemical compositions suggested a possible anti-inflammatory activity as the main biological property. Furthermore, the enantioselective analyses permitted to hypothesise that all these EOs are attractive for the insects of genus *Helicoverpa*, thanks to the extremely high enantiomeric excess of laevorotatory germacrene D.

## Figures and Tables

**Table 1:** Qualitative and quantitative compositions of *G. hallii*, *G. calyculisolvens*, and *G. azuayensis* EOs on two stationary phases of different polarity (5% phenyl methylpolysiloxane and polyethylene glycol).

N.	Compounds	5% Phenyl Methyl Polysiloxane									Polyethylene Glycol								
		LRI		*G. hallii*		*G. calyculisolvens*		*G. azuayensis*		Lit.	LRI		*G. hallii*		*G. calyculisolvens*		*G. azuayensis*		Lit.
		Calc.	Ref.	%	σ	%	σ	%	σ		Calc.	Ref.	%	σ	%	σ	%	σ	
1	heptanal	910	901	0.1	0.02	-	-	-	-	[15]	1158	1158	trace	-	-	-	-	-	[16]
2	**α-pinene**	935	932	33.6	2.39	11.2	2.59	4.5	1.42	[15]	1015	1015	31.5	2.38	11.0	2.41	4.1	1.14	[17]
3	α-fenchene	948	945	0.1	0.01	-	-	-	-	[15]	1074	1073	0.3	0.04	-	-	-	-	[18]
4	thuja-2,4(10)-diene	953	953	trace	-	-	-	-	-	[15]	-	-	-	-	-	-	-	-	-
5	sabinene	976	969	0.7	0.05	1.2	0.10	1.2	0.32	[15]	1113	1113	0.7	0.05	1.1	0.07	1.2	0.26	[19]
6	benzaldehyde	978	978	-	-	-	-	0.1	0.04	[15]	1514	1516	-	-	-	-	trace	-	[20]
7	β-pinene	980	979	0.3	0.02	0.3	0.02	0.2	0.11	[15]	1102	1102	o.t.p. 3	-	0.3	0.02	0.1	0.08	[21]
8	myrcene	994	988	0.2	0.02	0.4	0.03	0.6	0.10	[15]	1159	1159	0.2	0.01	0.5	0.02	0.5	0.06	[22]
9	2-pentyl furan	997	984	0.1	0.02	0.2	0.05	1.4	0.34	[15]	1229	1229	0.2	0.03	0.3	0.04	1.4	0.10	[23]
10	meta-mentha-1(7),8-diene	1003	1000	trace	-	-	-	-	-	[15]	-	-	-	-	-	-	-	-	-
11	α-phellandrene	1007	1002	-	-	0.9	0.08	-	-	[15]	1165	1164	-	-	0.7	0.02	-	-	[24]
12	(2*E*,4*E*)-heptadienal	1013	1005	trace	-	-	-	0.6	0.10	[15]	1463	1463	trace	-	-	-	o.t.p. 64	-	[25]
13	*n*-octanal	1015	998	-	-	-	-			[15]	1285	1286	-	-	-	-	0.2	0.07	[23]
14	α-terpinene	1021	1014	-	-	-	-	0.1	0.03	[15]	1168	1167	-	-	-	-	0.1	0.01	[26]
15	***p*** **-cymene**	1027	1020	0.1	0.01	-	-	-	-	[15]	1237	1237	trace	-	-	-	-	-	[27]
16	*o*-cymene	1028	1022	-	-	4.0	0.56	-	-	[15]	1238	1234	-	-	3.7	0.42	-	-	[28]
17	(2*E*,4*Z*)-heptadienal	1028	1013	-	-	-	-	0.6	0.09	[15]	1488	1480	-	-	-	-	0.6	0.18	[29]
18	limonene	1033	1024	trace	-	1.4	0.06	0.4	0.04	[15]	1187	1186	0.1	0.01	1.3	0.04	0.2	0.02	[30]
19	β-phellandrene	1031	1025	trace	-	-	-	-	-	[15]	1162	1161	trace	-	-	-	-	-	[31]
20	(*Z*)-β-ocimene	1042	1032	-	-	-	-	0.1	0.02	[15]	1232	1232	-	-	-	-	0.2	0.18	[32]
21	(*E*)-β-ocimene	1052	1044	trace	-	0.1	0.01	1.4	0.35	[15]	1247	1247	trace	-	0.1	0.01	1.4	0.18	[33]
22	γ-terpinene	1060	1054	trace	-	-	-	-	-	[15]	1221	1221	trace	-	-	-	-	-	[34]
23	benzene acetaldehyde	1061	1062	-	-	-	-	0.9	0.09	[15]	1639	1639	-	-	-	-	1.0	0.18	[35]
24	(2*E*)-octen-1-al	1072	1049	-	-	-	-	0.2	0.05	[15]	1421	1421	-	-	-	-	0.1	0.05	[36]
25	*cis*-linalool oxide (furanoid)	1078	1067	-	-	-	-	0.4	0.09	[15]	1442	1445	-	-	-	-	0.5	0.02	[37]
26	terpinolene	1086	1086	trace	-	-	-	-	-	[15]	1242	1239	trace	-	-	-	-	-	[38]
27	6-camphenone	1104	1095	-	-	0.20	0.01	-	-	[15]	1416	-	-	-	0.10	0.02	-	-	§
28	linalool	1110	1095	-	-	trace	-	1.3	0.06	[15]	1558	1560	-	-	trace	-	1.1	0.07	[39]
29	*n*-nonanal	1116	1100	0.3	0.02	0.4	0.03	0.8	0.27	[15]	1389	1389	0.2	0.01	0.3	0.04	0.4	0.08	[40]
30	γ-campholene aldehyde	1134	1122	0.1	0.02	-	-	-	-	[15]	1435	1439	trace	-	-	-	-	-	[41]
31	*trans*-pinocarveol	1147	1135	trace	-	-	-	-	-	[15]	-	-	-	-	-	-	-	-	-
32	(*E*)-epoxy-ocimene	1148	1137	trace	-	-	-	-	-	[15]	-	-	-	-	-	-	-	-	-
33	*trans*-verbenol	1154	1140	0.1	0.02	0.2	0.03	-	-	[15]	-	-	-	-	-	-	-	-	-
34	eucarvone	1166	1146	trace	-	0.2	0.02	-	-	[15]	-	-	-	-	-	-	-	-	-
35	*(*2*E)*-nonen-1-al	1173	1157	-	-	trace	-	0.2	0.03	[15]	1528	1528	-	-	trace	-	0.2	0.05	[42]
36	safranal	1176	1197	-	-	trace	-	-	-	[15]	-	-	-	-	-	-	-	-	-
37	*p*-mentha-1,5-dien-8-ol	1185	1166	-	-	-	-	0.1	0.03	[15]	1731	1738	-	-	-	-	0.2	0.02	[43]
38	terpinen-4-ol	1190	1174	0.1	0.02	0.2	0.02	0.4	0.02	[15]	1599	1599	0.5	0.02	0.5	0.14	0.4	0.11	[44]
39	*n*-dodecane	1200	1200	0.1	0.04	-	-	0.8	0.12	-	1200	1200	-	-	-	-	0.9	0.20	-
40	myrtenol	1204	1194	0.1	0.11	-	-	-	-	[15]	1740	1747	trace	-	-	-	-	-	[45]
41	myrtenal	1206	1195	-	-	0.3	0.05	-	-	[15]	1601	1601	-	-	0.3	0.02	-	-	[46]
42	α-terpineol	1207	1207	-	-	-	-	0.8	0.10	[15]	1698	1700	-	-	-	-	o.t.p. 93	-	[47]
43	verbenone	1209	1204	trace	-	0.3	0.06	-	-	[15]	-	-	-	-	-	-	-	-	-
44	*n*-decanal	1217	1201	0.1	0.03	0.3	0.02	1.1	0.27	[15]	1493	1495	trace	-	0.7	0.06	0.9	0.08	[48]
45	nerol	1237	1227	-	-	-	-	0.1	0.01	[15]	1805	1808	-	-	-	-	o.t.p. 101	-	[49]
46	thymol, methyl ether	1238	1232	-	-	0.2	0.2	-	-	[15]	1543	1555	-	-	0.3	0.05	-	-	[50]
47	cumin aldehyde	1256	1238	-	-	trace	-	-	-	[15]	1726	1738	-	-	trace	-	-	-	[51]
48	geranial	1262	1264	-	-	0.2	0.02	-	-	[15]	1720	1718	-	-	trace	-	-	-	[52]
49	geraniol	1263	1249	-	-	-	-	1.1	0.06	[15]	1856	1856	-	-	-	-	1.1	0.12	[53]
50	(2*E*)-decanal	1275	1260	0.1	0.01	0.5	0.02	0.1	0.04	[15]	1635	1638	0.4	0.02	0.7	0.03	0.1	0.02	[54]
51	nonanoic acid	1293	1267	-	-	-	-	0.1	0.01	[15]	2189	2190	-	-	-	-	0.2	0.11	[55]
52	*n*-tridecane	1300	1300	-	-	-	-	0.1	0.02	-	1300	1300	-	-	-	-	0.1	0.04	-
53	*trans*-pinocarvyl acetate	1303	1298	-	-	0.1	0.01	-	-	[15]	1638	1641	-	-	0.1	0.03	-	-	[56]
54	(2*E*,4*Z*)-decadienal	1309	1292	trace	-	-	-	0.1	0.02	[15]	1760	1759	trace	-	-	-	0.2	0.05	[57]
55	carvacrol	1317	1315	-	-	2.0	0.03	-	-	[58]	2186	2186	-	-	2.7	0.18	-	-	[59]
56	undecanal	1318	1305	-	-	-	-	0.4	0.07	[15]	1602	1598	-	-	-	-	0.5	0.09	[60]
57	*p*-vinylguaiacol	1323	1309	1.0	0.32	2.2	0.35	-	-	[15]	2182	2182	1.3	0.25	1.9	0.31	-	-	[61]
58	δ-elemene	1330	1335	0.1	0.02	-	-	-	-	[15]	-	-	-	-	-	-	-	-	-
59	unidentified (MW = 150)	1332	-	-	-	-	-	0.9	0.04	-	1801	-	-	-	-	-	0.7	0.11	-
60	(2*E*,4*E*)-decadienal	1334	1334	-	-	-	-			[15]	1801	1800	-	-	-	-			[62]
61	α-cubebene	1347	1348	trace	-	0.1	0.01	-	-	[15]	1416	1420	0.5	0.03	trace	-	-	-	[63]
62	neryl acetate	1367	1359	-	-	0.3	0.02	-	-	[15]	1682	1680	-	-	0.1	0.01	-	-	[49]
63	α-ylangene	1374	1373	0.5	0.02	-	-	-	-	[15]	1447	1450	0.5	0.05	-	-	-	-	[64]
64	**α-copaene**	1377	1374	-	-	3.6	0.43	0.7	0.17	[15]	1469	1471	-	-	3.7	0.42	0.7	0.17	[65]
65	β-bourbonene	1384	1387	-	-	0.2	0.01	-	-	[15]	1497	1490	-	-	0.2	0.02	-	-	[66]
66	α-isocomene	1386	1387	-	-	-	-	0.1	0.06	[15]	1500	1510	-	-	-	-	0.2	0.03	[65]
67	geranyl acetate	1386	1379	-	-	2.8	0.29	-	-	[15]	1716	1713	-	-	2.9	0.25	-	-	[50]
68	β-cubebene	1386	1387	0.4	0.02	1.0	0.04	-	-	[15]	1510	1508	trace	-	0.8	0.07	-	-	[67]
69	β-elemene	1389	1389			0.2	0.01	-	-	[15]	1554	1559	trace	-	-	-	-	-	[68]
70	(*E*)-β-damascenone	1389	1383	-	-	-	-	1.7	0.31	[15]	1808	1803	-	-	-	-	o.t.p. 101	-	[69]
71	decanoic acid	1391	1364	-	-	-	-			[15]	2243	2244	-	-	-	-	1.6	0.56	[70]
72	*n*-tetradecane	1400	1400	trace	-	0.1	0.01	0.6	0.02	-	1400	1400	-	-	0.1	0.01	0.5	0.19	-
73	α-gurjunene	1408	1409	0.4	0.02	0.3	0.04	0.2	0.04	[15]	1506	1507	-	-	0.3	0.02	0.1	0.01	[71]
74	methyl eugenol	1416	1403	-	-	0.6	0.05	-	-	[15]	-	-	-	-	-	-	-	-	-
75	**(*E*)-β-caryophyllene**	1418	1417	6.2	0.27	8.1	0.33	1.2	0.25	[15]	1551	1550	6.4	0.20	8.3	0.46	0.7	0.17	[72]
76	dodecanal	1420	1408	-	-	-	-			[15]	1700	1698	-	-	-	-	o.t.p. 93	-	[73]
77	unidentified (MW = 190)	1420	-	-	-	-	-			-	2115	-	-	-	-	-	0.6	0.10	-
78	β-duprezianene	1421	1421	-	-	-	-			[15]	2211	-	-	-	-	-	o.t.p. 126	-	§
79	β-copaene	1433	1430	-	-	0.2	0.03	0.1	0.01	[15]	1549	1550	-	-	trace	-	0.2	0.04	[64]
80	allo-aromadendrene	1446	1437	0.1	0.01	0.1	0.02	-	-	[15]	-	-	0.3	0.02	-	-	-	-	[74]
81	α-humulene	1452	1452	0.7	0.03	1.1	0.07	1.1	0.18	[15]	1643	1645	0.8	0.06	1.1	0.01	1.2	0.18	[75]
82	α-guaiene	1455	1453	0.9	0.19	-	-	-	-	[15]	1586	1583	1.1	0.05	-	-	-	-	[76]
83	9-*epi*-(*E*)-caryophyllene	1463	1464	-	-	-	-	0.2	0.03	[15]	1618	1630	-	-	-	-	trace	-	[77]
84	*cis*-cadina-1(6),4-diene	1465	1461	-	-	0.2	0.07	-	-	[15]	-	-	-	-	-	-	-	-	-
85	4,5-di-*epi*-aristolochene	1466	1471	0.2	0.05	-	-	-	-	[15]	-	-	o.t.p. 82	-	-	-	-	-	-
86	β-acoradiene	1474	1469	0.7	0.16	-	-	-	-	[15]	1691	1693	0.2	0.02	-	-	-	-	[78]
87	*trans*-cadina-1(6),4-diene	1479	1475	-	-	0.3	0.17	-	-	[15]	1613	-	-	-	0.5	0.12	-	-	§
88	γ-muurolene	1480	1478	-	-	-	-	0.2	0.07	[15]	1665	1665	-	-	-	-	0.1	0.09	[79]
89	**germacrene D**	1486	1480	35.7	1.02	20.8	0.65	14.1	2.35	[15]	1684	1684	38.3	1.63	22.0	0.23	12.4	1.69	[80]
90	(*E*)-β-ionone	1490	1487	-	-	-	-	1.9	0.84	[15]	1925	1926	-	-	-	-	1.6	0.18	[81]
91	valencene	1491	1496	-	-	0.2	0.02	-	-	[15]	-	-	-	-	-	-	-	-	-
92	α-zingiberene	1495	1493	-	-	-	-	0.7	0.14	[15]	1720	1721	-	-	-	-	0.6	0.22	[82]
93	**bicyclogermacrene**	1500	1500	3.8	0.11	1.2	0.05	2.6	0.54	[15]	1708	1711	4.0	0.17	1	0.03	3.0	0.66	[83]
94	α-muurolene	1501	1500	0.2	0.03	1.2	0.05	-	-	[15]	1705	1700	0.2	0.01	1.1	0.02	-	-	[84]
95	β-himachalene	1504	1500	trace	-	-	-	-	-	[15]	1704	1704			-	-	-	-	[85]
96	(*E*,*E*)-α-farnesene	1509	1505	-	-	trace	-	2.4	1.03	[15]	1742	1745	-	-	-	-	2.5	1.01	[49]
97	germacrene A	1511	1508	-	-	trace	-	-	-	[15]	1737	1738	-	-	trace	-	-	-	[86]
98	γ-cadinene	1515	1513	0.3	0.07	0.5	0.04	-	-	[15]	1666	1666	0.1	0.02	0.5	0.04	-	-	[87]
99	δ-amorphene	1519	1511	1.2	0.12	-	-	-	-	[15]	1707	1710	1.3	0.12	-	-	-	-	[88]
100	δ-cadinene	1522	1522	-	-	2.4	0.16	-	-	[15]	1713	1708	-	-	2.0	0.16	-	-	[89]
101	**tridecanal**	1522	1509	-	-	-	-	6.4	0.22	[15]	1810	1811	-	-	-	-	6.2	0.24	[60]
102	*trans*-cadina-1,4-diene	1535	1533	trace	-	-	-	-	-	[15]	-	-	-	-	-	-	-	-	-
103	*cis*-muurol-5-en-4-β-ol	1554	1550	-	-	0.1	0.03	-	-	[15]	-	-	-	-	-	-	-	-	-
104	(*E*)-nerolidol	1565	1561	0.1	0.02	0.2	0.08	-	-	[15]	2003	2005	0.1	0.02	0.2	0.02	-	-	[73]
105	elemicin	1565	1555	-	-	1.9	1.74	-	-	[15]	2212	2214	-	-	1.5	0.09	-	-	[90]
106	unidentified (MW = 220)	1577	-	-	-	-	-	0.4	0.05	-	1891	-	-	-	-	-	0.2	0.03	-
107	β-copaen-4-α-ol	1580	1590	-	-	0.2	0.02	-	-	[15]	2226	-	-	-	0.1	0.01	-	-	§
108	**germacrene D-4-ol**	1581	1574	0.5	0.18	8.4	0.49	-	-	[15]	2037	2038	0.6	0.07	8.6	0.43	-	-	[91]
109	**spathulenol**	1583	1577	0.6	0.14	-	-	7.8	1.26	[15]	2121	2121	-	-	-	-	7.1	1.08	[92]
110	unidentified (mw: 220)	1591	-	-	-	0.9	0.28	-	-	-	2074	-	-	-	1.1	0.11	-	-	-
111	caryophyllene oxide	1591	1582	0.5	0.15	1.8	0.26	3.1	0.16	[15]	1955	1955	0.2	0.07	1.8	0.22	1.9	0.16	[89]
112	unidentified (MW = 220)	1595	-	-	-	-	-			-	1984	-	-	-	-	-	0.3	0.09	-
113	unidentified (MW = 220)	1596	-	-	-	-	-			-	2247	-	-	-	-	-	0.5	0.16	-
114	*n*-hexadecane	1600	1600	-	-	-	-	o.t.p. 111	-	-	1600	1600	-	-	-	-	0.2	0.04	-
115	guaiol	1605	1600	-	-	0.6	0.06	-	-	[15]	2054	2064	-	-	0.4	0.01	-	-	[93]
116	ledol	1610	1602	0.4	0.04	-	-	-	-	[15]	2018	2016	0.3	0.03	-	-	-	-	[94]
117	humulene epoxide II	1621	1608	-	-	-	-	1.1	0.07	[15]	2012	2024	-	-	-	-	0.6	0.10	[95]
118	tetradecanal	1623	1611	-	-	-	-			[15]	1915	1919	-	-	-	-	0.5	0.04	[96]
119	junenol	1627	1618	trace	-	-	-	0.5	0.13	[15]	2028	2028	trace	-	-	-	0.2	0.06	[95]
120	unidentified (MW = 220)	1629	-	-	-	-	-	1.1	0.17	-	2241	-	-	-	-	-	o.t.p. 71	-	-
121	himachalol	1641	1652	-	-	0.10	0.04	-	-	[15]	-	-	-	-	-	-	-	-	-
122	*allo*-aromadendrene epoxide	1641	1639	0.1	0.02	0.1	0.03	-	-	[15]	2092	2095	0.1	0.02	0.1	0.01	-	-	[97]
123	epi-α-cadinol	1650	1638	0.3	0.05	0.7	0.11	-	-	[15]	2126	2126	0.3	0.04	0.7	0.08	-	-	[38]
124	*epi*-α-muurolol	1652	1640	0.5	0.09	0.7	0.12	0.4	0.06	[15]	2181	2182	0.9	0.06	0.8	0.10	0.5	0.14	[98]
125	*α*-muurolol	1654	1644	0.1	0.08	0.4	0.13	0.5	0.12	[15]	2166	2165	0.3	0.05	0.4	0.03	0.5	0.14	[99]
126	α-cadinol	1664	1652	0.5	0.18	1.9	0.23	1.2	0.07	[15]	2213	2211	o.t.p. 124	-	1.8	0.29	2.0	0.13	[100]
127	unidentified (MW = 220)	1673	-	-	-	-	-	0.8	0.17	-	2067	-	-	-	-	-	0.6	0.07	-
128	*epi*-zizanone	1674	1668	0.1	0.02	-	-	-	-	[15]	-	-	-	-	-	-	-	-	-
129	14-hydroxy-9-*epi*-(*E*)-caryophyllene	1684	1668	0.5	-	-	-	-	-	[15]	2066	-	0.6	0.18	-	-	-	-	§
130	khusinol	1692	1679	0.1	0.02	0.4	0.04	-	-	[15]	2237	-	-	-	0.4	0.02	-	-	§
131	*n*-heptadecane	1700	1700	-	-	-	-	0.2	0.08	-	1700	1700	-	-	-	-	0.1	0.07	-
132	amorpha-4,9-dien-2-ol	1701	1700	0.1	0.04	-	-	0.9	0.27	[15]	2263	-	0.1	0.03	-	-	1.2	0.16	§
133	*n*-pentadecanal	1725	1724	-	-	0.5	0.02	2.7	0.07	[15]	2023	2020	-	-	0.5	0.04	2.9	0.16	[31]
134	β-acoradienol	1742	1762	trace	-	-	-	-	-	[15]	-	-	-	-	-	-	-	-	-
135	unidentified (MW = 220)	1781	-	-	-	-	-	0.8	0.12	-	2231	-	-	-	-	-	0.6	0.19	-
136	unidentified (MW = 220)	1784	-	-	-	-	-	0.5	0.03	-	2268	-	-	-	-	-	0.2	0.05	-
137	avocadynofuran	1784	1780	-	-	0.3	0.09			[15]	-	-	-	-	-	-	-	-	-
138	1-octadecene	1795	1789	-	-	0.1	0.01	-	-	[15]	1835	1823	-	-	0.4	0.04	-	-	[101]
139	*n*-octadecane	1800	1800	trace	-	trace	-	0.4	0.11	-	1800	1800	0.1	0.02	trace	-	0.2	0.11	-
140	14-hydroxy-δ-cadinene	1805	1803	trace	-	-	-	-	-	[15]	-	-	-	-	-	-	-	-	-
141	*n*-hexadecanal	1834	1830	0.1	0.02	0.1	0.02	0.8	0.22	[15]	2134	2137	-	-	-	-	1.1	0.04	[102]
142	6,10,14-trimethyl-2-pentadecanone	1856	1855	-	-	-	-	0.9	0.08	[15]	2127	2125	-	-	-	-	0.6	0.06	[103]
143	1-nonadecene	1893	1895	-	-	0.1	0.01	-	-	[15]	1936	1938	-	-	0.1	0.02	-	-	[104]
144	*n*-nonadecane	1900	1900	trace	-	0.5	0.01	0.2	0.03	-	1900	1900	trace	-	0.8	0.02	trace	-	-
145	(5*E*,9*E*)-farnesyl acetone	1925	1913	-	-	-	-	0.9	0.06	[15]	2244	-	-	-	-	-	1.1	0.22	§
146	*n*-heptadecanal	1935	1930	trace	-	0.1	0.01	1.5	0.10	[15]	2219	-	-	-	-	-	1.6	0.42	§
147	methyl hexadecanoate	1938	1921	-	-	-	-	0.2	0.02	[15]	2188	2191	-	-	-	-	0.4	0.17	[105]
148	unidentified (MW = 262)	1978	-	-	-	-	-	0.6	0.07	-	2235	-	-	-	-	-	trace	-	-
149	1-eicosene	1996	1987	-	-	0.2	0.01	-	-	[15]	2037	2047	-	-	0.4	0.01	-	-	[106]
150	*n*-eicosane	2001	2000	-	-	trace	-	0.1	0.02	-	2000	2000	-	-	-	-	0.1	0.06	-
151	*n*-octadecanal	2034	2033	-	-	-	-	0.2	0.02	[15]	2359	2357	-	-	-	-	0.2	0.04	[107]
152	unidentified (MW = 220)	2085	-	-	-	-	-	0.6	0.10	-	2294	-	-	-	-	-	0.5	0.30	-
153	1-heneicosene	2093	2098	-	-	0.1	0.01	-	-	[108]	2137	2127	-	-	0.2	0.02	-	-	[109]
154	unidentified (MW = 294)	2096	-	-	-	-	-	1.1	0.20	-	2376	-	-	-	-	-	1.7	0.15	-
155	*n*-heneicosane	2101	2100	0.1	0.07	0.4	0.06	0.4	0.01	-	2100	2100	trace	-	0.3	0.02	0.5	0.06	-
156	(*Z*)-phytol	2120	2114	-	-	-	-	2.5	0.53	[53]	2362	-	-	-	-	-	2.9	0.90	§
157	unidentified (MW = 355)	2152	-	-	-	-	-	0.4	0.05	-	2323	-	-	-	-	-	0.2	0.10	-
158	1-docosene	2196	2189	-	-	0.2	0.03	0.1	0.01	[15]	2289	-	-	-	-	-	0.7	0.02	§
159	*n*-docosane	2200	2200	-	-	trace	-	0.1	0.01	-	2200	2200	-	-	0.2	0.02	0.1	0.06	-
160	*n*-eicosanal	2237	2229	-	-	-	-	0.1	0.01	[110]	2575	2571	-	-	-	-	0.2	0.02	[111]
161	1-tricosene	2295	2296	trace	-	0.1	0.03	trace	-	[108]	2346	-	trace	-	-	-	0.5	0.05	§
162	*n*-tricosane	2300	2300	-	-	0.5	0.06	0.4	0.03	-	2300	2300	-	-	0.3	0.06	0.1	0.01	-
163	1-tetracosene	2396	2396	-	-	0.1	0.04	1.5	0.15	[108]	2467	-	-	-	0.2	0.02	0.4	0.15	§
164	*n*-tetracosane	2400	2400	-	-	0.1	0.03			-	2400	2400	-	-	trace	-	1.1	0.17	-
165	*n*-docosanal	2440	2434	-	-	-	-	0.5	0.25	[110]	2425	-	-	-	-	-	0.4	0.05	§
166	1-pentacosene	2496	2496	-	-	0.2	0.01	1.0	0.07	[108]	2476	2488	-	-	0.2	0.02	1.1	0.27	[63]
167	*n*-pentacosane	2500	2500	-	-	0.2	0.01	1.0	0.10	-	2500	2500	-	-	0.3	0.07	1.1	0.19	-
168	1-hexacosene	2596	2596	-	-	0.1	0.07	1.4	0.21	[112]	2551	-	-	-	0.5	0.06	1.6	0.29	§
169	*n*-hexacosane	2600	2600	-	-	0.1	0.07	-	-	-	2600	2600	-	-	0.3	0.04	-	-	-
	monoterpenes			35.0		19.5		8.5					32.8		18.7		7.8		
	oxygenated monoterpenoids			0.4		7.0		4.2					0.5		7.0		3.3		
	sesquiterpenes			52.4		43.9		25.3					55.0		43.4		21.7		
	oxygenated sesquiterpenoids			4.4		16.8		20.6					3.5		16.4		18.0		
	others			1.0		7.7		35.6					0.9		8.3		38.5		
	total			93.2		94.9		94.2					92.7		93.8		89.3		

LRI—linear retention index; calc.—calculated LRI; ref.—LRI from literature; lit.—reference literature; %—percent amount by weight; σ—standard deviation; trace—<0.1%; o.t.p.—overlapped to peak; MW—molecular weight; §—identified only by MS. The number and name of major compounds (≥3.0% on at least one column in at least one EO) are written in bold.

**Table 2:** Enantioselective analyses of *G. hallii*, *G. calyculisolvens*, and *G. azuayensis* EOs on 2,3-diacetyl-6-*tert*-butyldimethylsilyl-β-cyclodextrin and 2,3-diethyl-6-*tert*-butyldimethylsilyl-β-cyclodextrin chiral selectors.

Chiral Selector	Enantiomers	LRI	*G. hallii*		*G. calyculisolvens*		*G. azuayensis*	
			Distribution (%)	ee (%)	Distribution (%)	ee (%)	Distribution (%)	ee (%)
DAC	(1*S*,5*S*)-(–)-α-pinene	926	100.0	100.0	100.0	100.0	100.0	100.0
DAC	(1*R*,5*R*)-(+)-α-pinene	928	-		-		-	
DET	(1*R*,5*R*)-(+)-β-pinene	949	9.1	81.9	9.0	82.0	69.9	39.7
DET	(1*S*,5*S*)-(–)-β-pinene	960	90.9		91.0		30.1	
DET	(1*R*,5*R*)-(+)-sabinene	977	21.6	56.7	47.5	5.1	92.9	85.9
DET	(1*S*,5*S*)-(–)-sabinene	992	78.4		52.5		7.1	
DET	(*R*)-(–)-α-phellandrene	1022	-	-	100.0	100.0	-	-
DET	(*S*)-(+)-α-phellandrene	1025	-		-		-	
DET	(*R*)-(–)-β-phellandrene	1052	100.0	100.0	-	-	-	-
DET	(*S*)-(+)-β-phellandrene	1063	-		-		-	
DET	(*S*)-(–)-limonene	1059	60.8	21.6	94.1	88.2	-	-
DET	(*R*)-(+)-limonene	1075	39.2		5.9		-	
DET	(*R*)-(–) *cis*-linalool oxide (furanoid)	1099	-	-	-	-	30.8	38.4
DET	(*S*)-(+) *cis*-linalool oxide (furanoid)	1104	-		-		69.2	
DET	(*R*)-(–)-linalool	1181	-	-	-	-	48.4	3.3
DET	(*S*)-(+)-linalool	1194	-		-		51.6	
DET	(*S*)-(−)-α-terpineol	1300	-	-	-	-	39.4	21.2
DET	(*R*)-(+)-α-terpineol	1313	-		-		60.6	
DET	(1*R*,2*S*,6*S*,7*S*,8*S*)-(–)-α-copaene	1321	-	-	-	100.0	-	100.0
DET	(1*S*,2*R*,6*R*,7*R*,8*R*)-(+)-α-copaene	1323	-		100.0		100.0	
DAC	(*R*)-(–)-terpinen-4-ol	1338	66.7	33.4	43.5	14.0	100.0	-
DAC	(*S*)-(+)-terpinen-4-ol	1375	33.3		57.5		-	
DET	(*R*)-(+)-germacrene D	1459	-	100.0	3.1	93.8	1.1	97.8
DET	(*S*)-(–)-germacrene D	1467	100.0		96.9		98.9	

DAC—2,3-diacetyl-6-*tert*-butyldimethylsilyl-β-cyclodextrin; DET—2,3-diethyl-6-*tert*-butyldimethylsilyl-β-cyclodextrin; LRI—linear retention index; %—peak area percent; ee—enantiomeric excess.

## Data Availability

The datasets presented in this article are not readily available because they are part of an ongoing study. Requests to access the datasets should be directed to the corresponding author.
